# Predicting Heart Rate at the Anaerobic Threshold Using a Machine Learning Model Based on a Large-Scale Population Dataset

**DOI:** 10.3390/jcm14010021

**Published:** 2024-12-24

**Authors:** Atsuko Nakayama, Tomoharu Iwata, Hiroki Sakuma, Kunio Kashino, Hitonobu Tomoike

**Affiliations:** 1 Department of Cardiovascular Medicine, Sakakibara Heart Institute, Tokyo 183-0003, Japan; 2 Department of Cardiovascular Medicine, University of Tokyo, Tokyo 113-8654, Japan; 3 NTT Communication Science Laboratories, NTT Corporation, Kanagawa-ken 243-0198, Japan; 4 NTT Basic Research Laboratories, NTT Corporation, Kanagawa-ken 243-0198, Japan

**Keywords:** cardiac rehabilitation, target exercise heart rate, machine learning, gradient boosting, feature selection

## Abstract

**Background/Objectives:** For effective exercise prescription for patients with cardiovascular disease, it is important to determine the target heart rate at the level of the anaerobic threshold (AT-HR). The AT-HR is mainly determined by cardiopulmonary exercise testing (CPET). The aim of this study is to develop a machine learning (ML) model to predict the AT-HR solely from non-exercise clinical features. **Methods:** From consecutive 21,482 cases of CPET between 2 February 2008 and 1 December 2021, an appropriate subset was selected to train our ML model. Data consisted of 78 features, including age, sex, anthropometry, clinical diagnosis, cardiovascular risk factors, vital signs, blood tests, and echocardiography. We predicted the AT-HR using a ML method called gradient boosting, along with a rank of each feature in terms of its contribution to AT-HR prediction. The accuracy was evaluated by comparing the predicted AT-HR with the target HRs from guideline-recommended equations in terms of the mean absolute error (MAE). **Results:** A total of 8228 participants included healthy individuals and patients with cardiovascular disease and were 62 ± 15 years in mean age (69% male). The MAE of the AT-HR by the ML-based model was 7.7 ± 0.2 bpm, which was significantly smaller than those of the guideline-recommended equations; the results using Karvonen formulas with the coefficients 0.7 and 0.4 were 34.5 ± 0.3 bpm and 11.9 ± 0.2 bpm, respectively, and the results using simpler formulas, rest HR + 10 and +20 bpm, were 15.9 ± 0.3 and 9.7 ± 0.2 bpm, respectively. The feature ranking method revealed that the features that make a significant contribution to AT-HR prediction include the resting heart rate, age, N-terminal pro-brain natriuretic peptide (NT-proBNP), resting systolic blood pressure, highly sensitive C-reactive protein (hsCRP), cardiovascular disease diagnosis, and β-blockers, in that order. Prediction accuracy with the top 10 to 20 features was comparable to that with all features. **Conclusions:** An accurate prediction model of the AT-HR from non-exercise clinical features was proposed. We expect that it will facilitate performing cardiac rehabilitation. The feature selection technique newly unveiled some major determinants of AT-HR, such as NT-proBNP and hsCRP.

## 1. Introduction

Cardiac rehabilitation (CR) is an evidence-based, class I, guideline-recommended secondary prevention strategy for many cardiovascular conditions among international guidelines [1,2,3]. Exercise assessment and prescription based on cardiopulmonary exercise testing (CPET) are core components of CR programs [4,5,6]. Improvements in cardiorespiratory fitness through long-term CR reduce rates of hospitalization and mortality in apparently healthy and clinical populations [7,8,9,10]. Despite strong evidence of its effectiveness, the participation rate in CR remains low in many countries: 24% of Medicare fee-for-service beneficiaries eligible participated in outpatient CR in the USA [11], 4.2% in outpatient CR for patients with PCI or CABG in Japan [12], and from 0% to 94.9% attendance rates of those advised to participate in CR in European countries [13]. Many barriers impeding participation or attendance have been mentioned in promoting CR [13,14]. One of the practical circumstances related to low CR participation rates is that patients with cardiovascular disease need to know their own anaerobic threshold (AT) levels [15,16]. An individual estimate of AT is represented as oxygen consumption, heart rate (HR), or metabolic equivalents at the timing of AT during cardiopulmonary exercise testing (CPET).

The AT is defined as the level of physical exercise when the blood lactate level begins to rise and is also measurable non-invasively using the V-slope method [17,18]. Although CPET is the gold standard method needed for exercise prescription, this laboratory test requires costly equipment, space for placement, significant time to complete the testing, and highly trained professionals to administer and examine. When CPET is not available, the guideline of CR recommends the application of equations, such as the Karvonen formula or even simpler equations referencing age and/or resting HR [3,8,19,20].

Since the advent of CPET to quantitatively measure exercise performance, many predictive formulas have been proposed to predict peak oxygen consumption [8,19,20]. However, few have been reported regarding AT-HR prediction, especially for patients with various cardiovascular diseases [21]. To facilitate CR for the patients who need to improve cardiorespiratory fitness but cannot take CPET, an accurate prediction method of AT-HR is highly desirable.

There are several formulas and methods used to predict AT-HR or target HR during exercise. In 1957, Karvonen et al. reported the Karvonen formula, which relies on the estimation of the maximum heart rate (MHR), often calculated using the formula 220 minus age; AT-HR = MHR × k (coefficient) + rest HR [19]. The coefficient k is determined according to the subject’s physical robustness; for example, healthy people 0.7, cardiovascular patients 0.6, high-risk patients 0.4–0.5. While the Karvonen formula is still widely used for its convenience, several studies have shown that such a generic formula can be imprecise and may not accurately reflect an individual’s true maximum heart rate [20,21,22]. The aim of this study was to establish a robust AT-HR prediction method that can be applied to patients with various cardiovascular diseases of different severity.

## 2. Methods

### 2.1. Participants and Study Design

A single-center retrospective cross-sectional study was conducted on the results of the clinical tests, including CPET, carried out at the Sakakibara Heart Institute between February 2008 and December 2021. The age of the study population was 62 ± 15 years, with 69% of the participants being male. The subjects underwent a clinical assessment with the acquisition of age, sex, anthropometric variables, cardiovascular risk factors, clinical diagnosis, and medication use (Table 1). Echocardiography was conducted during the examination period of CPET. Routine blood tests were performed before the patients were enrolled in CR. CPET consisted of a ramp exercise using a cycle ergometer system (Fukuda ML-9000 (Fukuda Denshi, Tokyo, Japan) up to 2012 and a MLX-1000 CPX (Fukuda Denshi, Tokyo, Japan) device from 2013) along with expired gas analysis using a “breath-by-breath” respiratory mass spectrometry system (AERO MONITOR AS 300S, MINATO MEDICAL, Tokyo, Japan).

**Figure 1:**
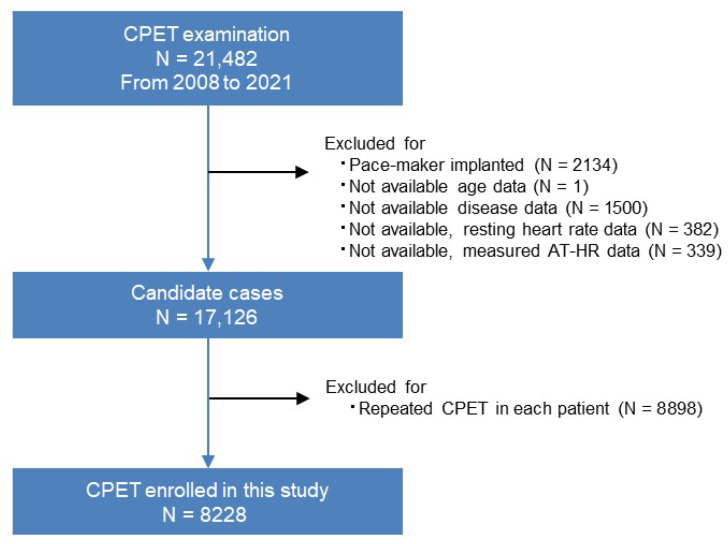
Flowchart of patient enrollment. From 2008 to 2021, a total of 21,491 CPETs were carried out. Among these, 10,915 were follow-up CPETs and 8228 were first-time CPETs for each patient. CPET: cardiopulmonary exercise testing.

Of the 21,482 cases, we selected 8228 cases for the present study, as shown in Figure 1 (flowchart of the study cases). We included not only healthy subjects but also patients with cardiovascular disease and/or cardiovascular risk factors and excluded participants who had a pacemaker implanted or no documentation of their age, disease data, resting heart rate, or heart rate at AT. The first examination outcome of CPET in each participant was included; therefore follow-up results were excluded. Among the included cases, echocardiography was performed on 4899 subjects (59.5% of the study population); details of medication were available in 80.0% of the population, items from blood tests were available in 40.2% to 78.2%, and life and family histories were available in 69.1% of the population (Table 1).

### 2.2. Prediction Algorithm

We used 78 patient characteristics as the prediction features from 51 clinical characteristics (Table 1). The outcome was predicted as AT-HR. We built a prediction model using a machine learning (ML) method called gradient boosting (GB) [23], which is based on an ensemble of decision trees and has been successfully applied to a wide range of problems. The study sample sets were repeatedly chosen by the cross-validation scheme, where the sets were randomly chosen by dividing the data into training (64% of patients), validation (16%), and test (20%) cohorts. Since missing data are acceptable in these processes, we included features that were not examined in all enrolled participants (Table 1). The model was trained using the training data, with the hyperparameters tuned with the validation data, and then the performance was evaluated using the test data.

### 2.3. Statistical Analysis

We identified two points that we needed to investigate statistically. The first was whether the cross-validation scheme was appropriately functioning. Cross-validation is a standard training and verification method widely used in the field of machine learning, but we thought it necessary to check it in the present problem and setup. The second was how accurate the proposed method was compared to the other existing methods. To address these issues, we took the following approach. First, as a basic verification of cross-validation, we checked whether the test set for each iteration was statistically unbiased by comparing the histograms of the features. Next, we measured the test accuracy for each test set in terms of the mean absolute error (MAE) between the estimated AT-HR and the actual CPX test results. If the MAE of the proposed method was smaller than that of other methods after taking into account the variation in the MAE, then it could be said that cross-validation was effective and also that the proposed method was significantly more accurate than the other methods. Therefore, we ran 30 experiments with different splits of training, validation, and test sets and averaged the MAE over those 30 test sets with their ± standard deviations (SD) to conduct t-tests. Statistical analysis was performed using Python 3.

### 2.4. Ranking of Features by Contribution to Prediction

We ranked features based on their contribution using a sequential forward feature selection method [24]. First, the model was trained using the training data set. Next, a selected feature set was initialized with an empty set. Then, for each step, an unselected feature was selected that achieved the lowest training MAE using the trained model when the feature was included in the selected feature set and unselected features’ values were set as missing. We repeated this step until all the features were selected. Features selected in earlier steps were considered to yield greater contribution.

## 3. Results

### 3.1. Characteristics of Study Population

Table 1 shows the patient background and clinical characteristics of the present study population, such as age, sex, height, weight, prevalence of cardiovascular risk factors (e.g., hypertension, dyslipidemia, diabetes, smoking status, and family history of coronary artery disease), clinical diagnosis, CPET outcomes, and echocardiographic findings.

As designed, cardiovascular comorbidities were prevalent, where occupations of ischemic diseases, heart surgeries (including coronary artery bypass grafting and valve replacements), heart failure, and aortic diseases were 21%, 6%, 3%, and 7%, respectively, along with heart diseases not related to CR insurance reinversement (43%). Clinically healthy subjects were also included (19%). Ejection fraction (EF) was 60 ± 11% (8–85%, median 62%), NT-proBNP was 750 ± 3122 (5—164,189, median 269) pg/mL, and the rate of β, αβ-blocker intake was 45% (Table 1).

### 3.2. Statistical Analysis on Test and Training + Validation Sets

As described in the Methods section, we confirmed that there was no statistical bias for each feature between the test and training-plus-validation sets in the cross-validation scheme. Namely, the correlation coefficients between the frequency distribution of the values of AT-HR, age, sex, height, weight, resting HR, resting systolic blood pressure, resting diastolic blood pressure, β-blocker intake, clinical diagnosis, smoking history, and NT-proBNP were 0.988, 0.987, 1.000, 0.997, 0.987, 0.999, 0.983, 0.998, 0.999, 0.999, 0.984, and 0.984, respectively. That is, the correlation between the distributions of feature values in the test set and the corresponding training-plus-validation sets was extremely high at 0.983 or above.

### 3.3. Evaluation of Predicted AT-HR

**Figure 2:**
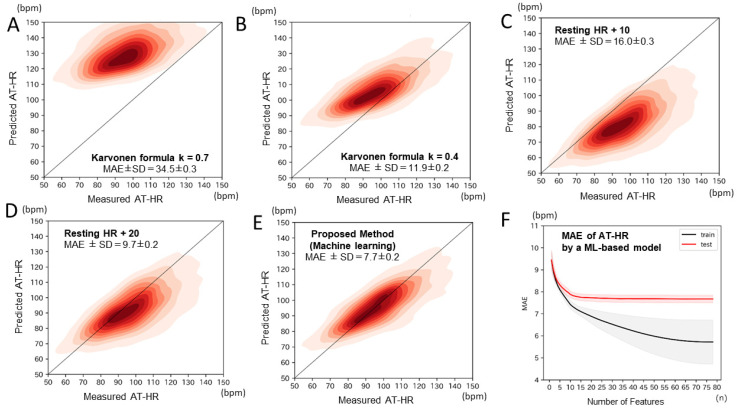
Predicted AT-HR and its accuracy. Comparison of the mean absolute errors (MAE) in predicting AT-HR using the Karvonen formula (coefficient, k = 0.7) (**A**), the Karvonen formula (coefficient, k = 0.4) (**B**), Resting HR + 10 (**C**), Resting HR + 20 (**D**), and machine learning model (**E**). (**F**) shows the relationship between the number of used features and the accuracy of the AT-HR prediction. The test curve (in red) shows that MAE decreases at first as the number of features increases, but after about 20 features, the decrease in errors plateaus. MAE: mean absolute error; AT-HR: anaerobic threshold heart rate.

The evaluation results are shown in Table 2 and Figure 2. We compared our model using 78 features with the Karvonen formula, which was calculated by ((220–Age)-Resting HR) k + Resting HR. In the Karvonen formula, k is usually chosen to be 0.7 for healthy people (Figure 2A) and 0.4 for patients with cardiovascular disease or heart failure (Figure 2B). Other simplified equations, such as (rest HR + 10) and (rest HR + 20), showed a MAE of 15.9 ± 0.3 and 9.7 ± 0.2 bpm, respectively (Figure 2C,D). Figure 2E shows the predicted AT-HR using the ML-based model. We confirmed that our model achieved the lowest MAE (7.7 ± 0.2 bpm), and it was significantly more accurate than the other existing equations (*p* < 0.001) (Table 2).

Figure 2F shows the MAE while increasing the number of employed features in the order of contribution to the AT-HR prediction. As the number of features increased, a corresponding decrease in MAE was observed. The inclusion of highly ranked features significantly reduced MAE, while lower-ranked features contributed minimally to improving the prediction performance. When the top 20 ranked features were utilized, the MAE was 7.8 ± 0.2, which was comparable to the MAE obtained when all the features were incorporated into the algorithm (7.7 ± 0.2).

### 3.4. Rank of Features in AT-HR Prediction

Table 3 presents the feature rankings, averaged across the 30 experiments. The results highlighted the most influential factors for predicting AT-HR. The top contributor was resting HR (Rank 1, Mean = 1.0, SD = 0.0, Correlation = 0.68), which had the strongest positive correlation coefficient with AT-HR, followed by age (Rank 2, Mean = 2.4, SD = 0.7, Correlation = −0.33), which negatively impacts AT-HR as older individuals tend to have lower AT-HR. Other significant factors included NT-proBNP (Rank 3, Mean = 4.4, SD = 1.7, Correlation = −0.03) and highly sensitive C-reactive protein (hsCRP; Rank 5, Mean = 5.9, SD = 2.3, Correlation = 0.004), which are often used for biomarkers related to heart failure and inflammation, respectively. Additionally, resting systolic blood pressure (Rank 4, Mean = 5.3, SD = 2.6, Correlation = 0.02) and diastolic blood pressure (Rank 10, Mean = 9.0, SD = 4.6, Correlation = 0.21), cardiovascular disease diagnosis (Rank 6, Mean = 6.9, SD = 2.2, Correlation = 0.18), β-blocker intake (Rank 7, Mean = 7.0, SD = 2.9, Correlation = −0.14), high-density lipoprotain (HDL) cholesterol levels (Rank 8, Mean = 11.0, SD = 1.9, Correlation = 0.14), and creatine kinase (CK) (Rank 11, Mean = 9.6, SD = 2.6, Correlation = 0.02) moderately contributed to predicting AT-HR.

## 4. Discussions

In this study, we showed that the ML approach was feasible to use in predicting AT-HR with clinically acceptable accuracy, namely with a MAE of less than 8 bpm. While the traditional AT-HR prediction formulas, such as those by Karvonen [19] and Nemoto [25], typically rely on factors such as resting HR, age, weight, height, and resting systolic blood pressure, our model identified additional important predictive factors, such as NT-proBNP, hsCRP, CK, and HDL cholesterol. The inclusion of these factors greatly improved the prediction accuracy. This is thanks to the fact that the ML technique can automatically identify key factors for AT-HR prediction. Interestingly, EF by echocardiographic test was ranked lower than we expected, indicating that AT-HR can be predicted with reasonable accuracy even without echocardiographic tests. This suggests the potential of simpler, non-invasive inputs for accurate AT-HR prediction in clinical settings.

### 4.1. Accuracy of Prediction

The advantage of the ML approach is that it can consider nonlinear relationships between the factors and the target. For example, factors such as β-blocker usage in cardiovascular patients and individual variations in cardiopulmonary functions can affect how the heart responds to a given exercise intensity in a complex manner and can lead to significant errors with linear models for estimating AT-HR [20]. Such advantages of the ML approach will also be advantageous in assessing treatment effects where multiple factors determine the clinical outcome in medical and health care procedures.

The presented prediction model was trained with the datasets containing a wide range of cases with respect to age (8–96 years old), subjects (healthy–severe heart failure), and other factors. To our knowledge, the employed datasets were not only the largest in size but also reliable in the sense that data were triple-checked by specialist cardiologists. We consider that these are the reasons why a high accuracy was achieved compared with other existing formulas.

### 4.2. Factors for Prediction

The strong factors that affect HR have been recognized as age [26], body mass index [27], body temperature [28], anxiety and stress [29], caffeine, nicotine, antiarrhythmia drugs [30], autonomic nervous activities, particularly sympathetic and parasympathetic activity [31], and arrhythmic states, including pacemaker implantation. In determining AT-HR, age and weight were important factors along with a cardinal influence of resting heart rate.

One of the new findings in the present study was that hsCRP, NT-proBNP, HDL-chol, and CK from blood tests were ranked in the top 10 determinants of AT-HR. NT-proBNP is one of the most important and sensitive indicators of cardiac overload status and has recently been recognized as not only an early marker but also as representing the severity of heart failure [32]. Therefore, NT-proBNP may serve as an important determinant of AT-HR due to its close relationship with cardiac output and oxygen delivery during exercise. Since HR is affected by cardiac disease, the fact that NT-proBNP, a straightforward indicator of cardiac status, was an important determinant of AT-HR was a remarkable finding in this study. Considering that hsCRP represents an inflammatory status, it is natural that the AT-HR is affected by hsCRP. Inflammation-induced sympathetic activation and reduced nitric oxide bioavailability impair vascular function, leading to heightened cardiovascular stress and elevated HR, particularly under exercise conditions [28].

HDL cholesterol is often reported to increase with aerobic exercise [33,34], suggesting that a lower resting HR may result in higher HDL cholesterol [35]. Since research on the relationship between CK and HR is still limited, we were not able to find any specific direct relationship in the literature. Such high contributions of NT-proBNP, HDL, CK, and hsCRP as predictors of AT-HR will require further analytical studies on the effective components of CR in ameliorating morbidity and mortality in age-related diseases, CVD, or cancers.

On the other hand, well-known parameters that represent cardiac pump performance such as EF (ranked 25th) or β-blocker (ranked 7th) were not ranked in the top five predictors. Although there have been studies on the relationship between EF and HR [36], it is difficult to investigate whether there is a direct correlation. β-blockers reduce the HR by blocking the action of adrenaline on β1 receptors [37]. By inhibiting the effects of these hormones, β-blockers decrease the electrical impulses that initiate each heartbeat. β-blockers, commonly prescribed for heart disease, were thought to impact HR. However, despite examining the dose of β-blockers, we found the effect on AT-HR to be relatively low. It is possible that these factors did not appear in the higher ranks because they were sufficiently represented by other factors.

For “factors for prediction”, new advanced parameters will be considered, such as genetic variants, socio-economic factors, or CPET-related exercise indexes. In the UK Biobank study, they found strong evidence that genetic variants associated with CRF and physical activity also influenced genetic expression in a small set of genes in the heart, artery, brain, lung, muscle, and adipose tissue [38]. As previously discussed, LVEF was reductive in the major determinants of AT-HR. However, cardiac pump performance is one of the major determinants of physical activity, and recent echocardiographic studies demonstrated that CR improves myocardial work indices, such as the global work index (GWI) or global constructive work (GCW); the GWI represents the area within the LV pressure-strain loop, and GCW indicates the positive work performed during isovolumic contraction time and systole (segment shortening) and negative work during isovlumic relaxation time (segment lengthening) [39]. When these parameters are added to the predictive equation, the accuracy of AT-HR estimation will improve.

### 4.3. Clinical Application of the Proposed Model and Its Effect on Health

**Figure 3:**
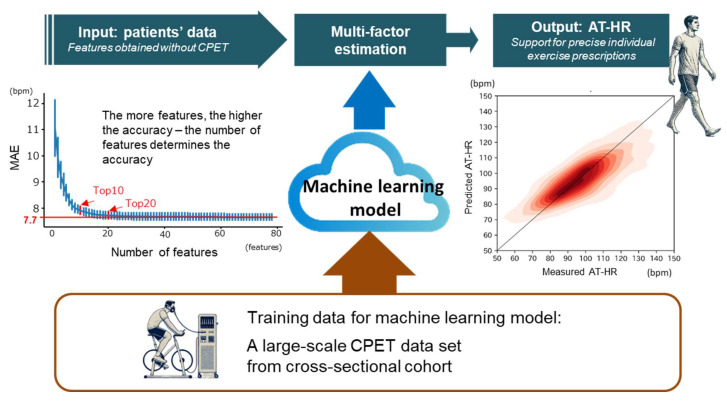
Impact of our machine-learning-based multi-factor AT-HR estimation. The model is trained with a CPET data set comprising a diverse cross-sectional cohort. Once trained, the model can predict individual AT-HR from various patient characteristics without actual CPET, allowing the patients to exercise with the knowledge of their AT-HR levels. CPET: cardiopulmonary exercise testing; AT-HR: anaerobic threshold heart rate.

Because physical inactivity was identified as an important risk factor for all-cause mortality [40], appropriate exercise is recommended as an interventional health activity [41]. Appropriate exercise is usually defined as aerobic exercise that has been reported to improve prognosis as well as to prevent cardiovascular events [42]. With the proposed AT-HR prediction system, city hospitals and private clinics without CPET equipment can obtain predicted AT-HR by entering basic information and providing aerobic exercise support to many patients based on the exercise prescription generated by the system (Figure 3). Even in large hospitals with CPET facilities, frequent testing tends to be difficult due to manpower and time constraints. Therefore, the system can also be used to frequently update exercise prescriptions in response to ever-changing functional states or performance levels of cardiovascular patients with various disease severities.

The present study was conducted on a commonly used personal computer and open-source software. Accordingly, this model will be applicable in low-resource settings such as in general practice.

AI-assisted AT-HR estimation enhances clinical practice by improving diagnostic accuracy, reducing observer variability, and enabling personalized cardiovascular care. Future directions involve integrating AI with other diagnostic modalities, advancing preventive strategies, and expanding applications in telemedicine and cardiac rehabilitation.

## 5. Limitations

Although this study used big data from a wide range of age groups, diseases, and healthy individuals, it was based on the Japanese population, and non-Japanese results need to be re-validated. Because data for model building and data for validation were separated, we believe the accuracy of AI learning was not overestimated, but validation of the results of exercise prescription by this system is necessary in the future.

## 6. Conclusions

Using a large number of patient data with various characteristics, we showed the effectiveness of a ML-based model for predicting AT-HR. By ranking features, we could predict AT-HR with a small number of patient characteristics. We revealed that NT-proBNP, hsCRP, CK, and HDL cholesterol were important features for predicting AT-HR, which were not focused on in the previous formulae.

## Figures and Tables

**Table 1:** Clinical characteristics.

Characteristics	Value (n = 8228)	Number
***Clinical characteristics***		
Age (years)	62 ± 15	8228
Male, n (%)	5686 (69)	8228
Hight (cm)	164 ± 9	7731
Weight (kg/m^2^)	63 ± 13	7710
Hypertension, n (%)	5142 (63)	8228
Dyslipidemia, n (%)	4694 (57)	8228
Diabetes, n (%)	1206 (15)	8228
Current smoking, n (%)	1083 (13)	5685
Ex-smoking, n (%)	1870 (23)	5685
Family history of CVD, n (%)	543 (7)	5685
***Cardiovascular disease***		
Ischemic heart disease		
AMI, n (%)	494 (6)	8228
AP, n (%)	698 (8)	8228
OMI, n (%)	9 (0)	8228
CABG, n (%)	576 (7)	8228
Cardiac surgery		
AVR, n (%)	281 (3)	8228
MVP, n (%)	147 (2)	8228
MVR, n (%)	44 (1)	8228
DVR, n (%)	22 (0)	8228
CHF, n (%)	227 (3)	8228
Aortic diseases, n (%)	560 (7)	8228
Normal subjects, n (%)	1574 (19)	8228
Non-rehabilitation heart disease, n (%)	3535 (43)	8228
***Cardiopulmonary exercise test***		
Rest HR (bpm) *	72 ± 12	8223
Peak VO_2_ (mL/kg/min)	20 ± 6	8220
%Peak VO_2_ (%)	81 ± 21	8220
AT VO_2_ (mL/kg/min)	13 ± 3	8219
ΔVE/ΔVCO_2_ slope	30 ± 7	7496
ΔVO_2_/ΔWR (mL/min/watt)	10 ± 2	8216
***Echocardiography***		
EF (%)	60 ± 11	4899
LVDd (mm)	47 ± 8	4899
LVDs (mm)	32 ± 7	4899
IVSth (mm)	10.3 ± 0.2	4899
PWth (mm)	9.9 ± 0.2	4899
***Blood tests***		
Albumin (g/dL)	4.3 ± 0.4	3310
Creatinine (mg/dL)	0.9 ± 0.5	6433
hsCRP (mg/dL)	0.4 ± 1.2	4009
HbA1c (NGSP)(%)	6.1 ± 0.8	3702
T-chol (mg/dL)	182 ± 38	5302
LDL-chlo (mg/dL)	102 ± 33	5568
HDL-chol (mg/dL)	52 ± 15	5442
TG (mg/dL)	145 ± 100	5629
NT-proBNP (pg/mL)	750 ± 3122	4523
***Medications***		
β, αβ- Blocker, n (%)	2974 (45)	6579
Dose of β, αβ- Blocker, (mg as Bisoprolol)	0.9 ± 1.6	6579
ACE inhibitor, n (%)	675 (8)	6579
ARB, n (%)	1140 (14)	6579
Ca blocker except for Verapamil/Diltiazem, n (%)	1439 (17)	6579
Ca blocker, Verapamil/Diltiazem, n (%)	314 (4)	6579
Statin, n (%)	2696 (33)	6579
Antiarrhythmic drugs, n (%)	337 (4)	6579
Digoxin, n (%)	69 (1)	6579

Data express as number and its study population or mean ± standard deviation. Abbreviations; CVD: cardiovascular disease, AMI: acute myocardial infarction, AP: angina pectoris, OMI; old myocardial infarction, CABG: coronary artery bypass grafting, AVR: aortic valve replacement, MVP: mitral valve plasty, DVR: double valve replacement, CHF: chronic heart failure, DA: aortic dissection, HR: heart rate, VO_2_: oxygen uptake, AT: anaerobic threshold, △: delta, WR: work rate (watt), EF: ejection fraction, LVDd: left ventricular end-diastolic diameter, LVDs: left ventricular end-systolic diameter, IVSth: intraventricular septum thickness, PWth: posterior wall thickness, hsCRP: high sensitive C-reactive protein, LDL: low-density lipoprotein, HDL: high-density lipoprotein, TG: triglyceride, NTproBNP: N-terminal pro-brain natriuretic peptide, ACE inhibitor: angiotensin-converting-enzyme inhibitor, ARB: angiotensin II receptor blocker. * Rest HR < 30 or 130 ≦ bpm (n = 5) were excluded because of error values.

**Table 2:** Tests of mean absolute errors predicting AT-HR.

	MAE	SD	Compared with ML Method by *t*-test
Resting HR + 10	15.9	0.3	<0.001
Resting HR + 20	9.7	0.2	<0.001
Resting HR + 30	10.1	0.1	<0.001
Karvonen (0.3)	8.6	0.2	<0.001
Karvonen (0.4)	11.9	0.2	<0.001
Karvonen (0.5)	18.4	0.2	<0.001
Karvonen (0.6)	26.2	0.3	<0.001
Karvonen (0.7)	34.5	0.3	<0.001
ML	7.7	0.2	-

AT-HR: anaerobic threshold heart rate. MAE: mean absolute error. Karvonen (k): Karvonen formula with coefficient k. ML: Machine Learning. ML method used all features.

**Table 3:** Rank of Features Contributing to AT-HR.

Rank	Feature	Demogram	Blood Tests	Echo	Medication	Mean	SD	Correlation Coefficient
1	Resting heart rate	〇				1	0	0.68
2	Age	〇				2.4	0.7	−0.33
3	NT−proBNP		〇			4.4	1.7	−0.03
4	Resting systolic blood pressure	〇				5.3	2.6	0.02
5	High sensitive C−reactive protein		〇			5.9	2.3	0.004
6	Cardiovascular diseases diagnosis	〇				6.9	2.2	0.18
7	β−blocker				〇	7	2.9	−0.14
8	Height	〇				8.8	2.4	0.04
9	Weight	〇				8.9	2.5	−0.01
10	Resting diastolic blood pressure	〇				9	4.6	0.21
11	Creatine kinase		〇			9.6	2.6	0.02
12	HDL cholesterol		〇			11	1.9	0.14
13	Triglycerides		〇			18	3.9	−0.01
14	Creatinine		〇			18.4	5.1	−0.09
15	Hemoglobin		〇			18.6	5.9	0.16
16	Blood glucose		〇			19.5	4.3	−0.06
17	Platelet		〇			19.6	5.4	0.1
18	Valsalva sinus diameter			〇		22.8	9.9	−0.08
19	White blood cell		〇			23.4	9	−0.01
20	Total cholesterol		〇			25	9	0.12
21	Interventricular septal thickness			〇		26.3	12.7	−0.2
22	Left atrial dimension			〇		27.9	11.1	−0.11
23	Hemoglobin A1c		〇			27.9	7.3	−0.06
24	Total protein		〇			28.1	9.3	0.1
25	Ejection fraction (MOD−sp4)			〇		28.3	7.4	−0.09
26	Calcium channel blockers				〇	28.5	12.3	−0.12
27	Albumin		〇			29.4	9.4	0.19
28	LDL cholesterol		〇			29.6	10.1	0.1
29	Tricuspid regurgitant pressure gradient			〇		32.2	8.2	−0.03
30	Ejection fraction (MOD−sp2)			〇		32.9	6.8	−0.07
31	Respiratory variability			〇		33.1	7.8	0.02
32	Smoking History	〇				33.6	14.7	−0.07
33	Maximum left atrial volume			〇		33.7	11.1	−0.03
34	Maximum left atrial volume index			〇		36.3	7.8	−0.05
35	End−diastolic volume (MOD−sp4)			〇		36.4	6	−0.01
36	Right ventricular area change rate			〇		37.1	10.4	−0.1
37	End−diastolic volume (Teich)			〇		37.2	8.9	0.01
38	End−systolic volume (Teich)			〇		39	12.2	0.05
39	End−systolic volume (MOD−sp4)			〇		41.5	9.4	0.04
40	Ascending aorta diameter			〇		42	14.8	−0.14
41	Right ventricular end−diastolic area			〇		42.5	11.6	0.04
42	End−systolic volume (MOD−sp2)			〇		43.8	7.8	0.03
43	Ejection fraction (Teich)			〇		44	8.5	−0.07
44	Tricuspid valve lateral systolic velocity			〇		44.5	10.1	−0.02
45	Left ventricular posterior wall thickness			〇		44.8	11.7	−0.16
46	End−systolic volume (MOD−bp)			〇		44.9	7.7	0.04
47	Left ventricular fractional shortening			〇		44.9	8.4	−0.07
48	Tricuspid annular plane systolic excursion			〇		45.2	9.8	−0.04
49	End−diastolic volume (MOD−sp2)			〇		45.2	10.3	−0.01
50	Tricuspid valve lateral early diastolic velocity			〇		46.3	12.3	0.16
51	Statins				〇	46.7	16.3	−0.09
52	Left ventricular outflow tract diameter			〇		46.7	13.9	0.06
53	Right ventricular end−systolic area			〇		50.2	11.5	0.06
54	Maximum inferior vena cava diameter			〇		51.1	9.3	0.06
55	Antiarrhythmic drugs				〇	55.3	15.3	−0.08
56	Aortic valve mean pressure gradient			〇		55.9	8.1	−0.13
57	Verapamil or Diltiazem				〇	56.4	16.5	−0.01
58	Aortic valve maximum pressure gradient			〇		57.1	5.4	−0.14
59	Digoxin				〇	57.1	19.6	0.02
60	Left ventricular end−diastolic diameter			〇		58	8.9	0.002
61	Diuretics				〇	58.2	14.2	−0.02
62	Sex	〇				59	8.8	−0.05
63	Antiplatelet drugs				〇	60.1	9.5	−0.11
64	Left ventricular end−systolic diameter			〇		61.1	7.1	0.04
65	Minimum inferior vena cava diameter			〇		61.3	7	0.02
66	Non−statin lipid−lowering drugs				〇	62.5	14	−0.02
67	Anticoagulants				〇	62.9	7.6	0
68	Angiotensin−converting enzyme inhibitors				〇	65.2	11.4	0.01
69	Aortic valve maximum flow velocity			〇		65.3	6.8	−0.14
70	Family medical history	〇				66.4	7.9	0.01
71	Antidiabetic drugs				〇	67	9.5	−0.03
72	Angiotensin receptor blockers				〇	67.4	9.1	−0.06
73	Right ventricular outflow tract diameter			〇		70.7	4	0.09
74	Sinotubular junction diameter			〇		72.4	4.3	0.01
75	Main pulmonary artery diameter			〇		73	4.3	0.11
76	Aortic valve annulus diameter			〇		73.1	4.7	0.08
77	Tricuspid valve E/e’ Ratio			〇		73.9	3.9	−0.11
78	Tricuspid valve E−wave velocity			〇		74.2	3.5	0.03

AT-HR: anaerobic threshold heart rate, NTproBNP: N-terminal pro-brain natriuretic peptide, HDL: high-density lipoprotein, LDL: low-density lipoprotein, MOD-sp: modified Simpson

## Data Availability

The datasets presented in this article are not readily available because the data are part of an ongoing study. Requests to access the datasets should be directed to Atsuko Nakayama, Sakakibara Heart Institute [anakaya@shi.heart.or.jp].

## Abbreviations

CR	cardiac rehabilitation
AT-HR	heart rate at the level of anaerobic threshold
CPET	cardiopulmonary exercise testing
ML	machine learning
